# Optimal multiparametric set-up modelled for best survival outcomes in palliative treatment of liver malignancies: unsupervised machine learning and 3 PM recommendations

**DOI:** 10.1007/s13167-020-00221-2

**Published:** 2020-08-10

**Authors:** Elisha Goldstein, Kristina Yeghiazaryan, Ashar Ahmad, Frank A. Giordano, Holger Fröhlich, Olga Golubnitschaja

**Affiliations:** 1 Machine learning research group, Department of Bioinformatics, Weizmann Institute, Rehovot, Israel; 2 State NRW-Israel program, Rheinische Friedrich-Wilhelms Universität Bonn, Bonn, Germany; 3 IT-Department, University Hospital Bonn, Rheinische Friedrich-Wilhelms Universität Bonn, Bonn, Germany; 4 AI & Data Science, Department of Bioinformatics, Fraunhofer Institute for Algorithms and Scientific Computing (SCAI), 53754 Sankt Augustin, Germany; 5 Bonn-Aachen International Centre for IT, Rheinische Friedrich-Wilhelms-Universität Bonn, 53115 Bonn, Germany; 6 Department of Radiation Oncology, University Hospital Bonn, Rheinische Friedrich-Wilhelms Universität Bonn, Bonn, Germany; 7 Predictive, Preventive and Personalised (3P) Medicine, Department of Radiation Oncology, University Hospital Bonn, Rheinische Friedrich-Wilhelms Universität Bonn, Venusberg-Campus 1, 53127 Bonn, Germany

**Keywords:** Predictive preventive personalised medicine (PPPM/3 PM), Liver diseases, Liver malignancy, Palliative medicine, Unsupervised machine learning, Multiparametric modelling, Patient stratification, Hepatocellular carcinoma, Colorectal cancer, Breast cancer, Prostate cancer, Metastasis, Liquid biopsy, Ex vivo, Circulating leucocytes, Multi-omics, Biomarker patterns, Multi-level diagnostics, Survival, Prognosis, Metalloproteinase, Comet assay, Calgranulin A, S100, Catalase, Superoxide-dismutase 2, SOD-2, Profilin, Rho A, Thioredoxin, Scavenger, Antioxidant compounds, ROS inhibition, Selective internal radiation therapy (SIRT), Trans-arterial chemo-embolisation (TACE), Individual outcomes, Covid-19, Viral infection, Hepatitis, Hypoxia, Inflammation, Detoxification, Impairments, Individualised patient profile, Redox status, Genoprotection, Phytochemicals, Redox-based therapy, Risk mitigation

## Abstract

Over the last decade, a rapid rise in deaths due to liver disease has been observed especially amongst young people. Nowadays liver disease accounts for approximately 2 million deaths per year worldwide: 1 million due to complications of cirrhosis and 1 million due to viral hepatitis and hepatocellular carcinoma. Besides primary liver malignancies, almost all solid tumours are capable to spread metastases to the liver, in particular, gastrointestinal cancers, breast and genitourinary cancers, lung cancer, melanomas and sarcomas. A big portion of liver malignancies undergo palliative care. To this end, the paradigm of the palliative care in the liver cancer management is evolving from “just end of the life” care to careful evaluation of all aspects relevant for the survivorship. In the presented study, an evidence-based approach has been taken to target molecular pathways and subcellular components for modelling most optimal conditions with the longest survival rates for patients diagnosed with advanced liver malignancies who underwent palliative treatments. We developed an unsupervised machine learning (UML) approach to robustly identify patient subgroups based on estimated survival curves for each individual patient and each individual potential biomarker. UML using consensus hierarchical clustering of biomarker derived risk profiles resulted into 3 stable patient subgroups. There were no significant differences in age, gender, therapy, diagnosis or comorbidities across clusters. Survival times across clusters differed significantly. Furthermore, several of the biomarkers demonstrated highly significant pairwise differences between clusters after correction for multiple testing, namely, “comet assay” patterns of classes I, III, IV and expression rates of calgranulin A (S100), SOD2 and profilin—all measured ex vivo in circulating leucocytes. Considering worst, intermediate and best survival curves with regard to identified clusters and corresponding patterns of parameters measured, clear differences were found for “comet assay” and S100 expression patterns. In conclusion, multi-faceted cancer control within the palliative care of liver malignancies is crucial for improved disease outcomes including individualised patient profiling, predictive models and implementation of corresponding cost-effective risks mitigating measures detailed in the paper. The “proof-of-principle” model is presented.

## Introduction

### Multi-factorial liver disease—healthcare and economic burden

Detoxification function by the liver is central for all physiological processes in humans. Chronic liver pathologies are life-threatening conditions with a highly heterogeneous clinical picture and large spectrum of potential consequences. Viral infections, fat liver, liver cirrhosis and primary liver malignancies altogether create a considerable subpopulation of chronically diseased people. A reciprocal relationship between infection disorders and liver pathologies has been demonstrated as follows: Hepatitis B and C is the frequent cause of inflammatory liver diseases on one hand, and on the other hand, liver dysfunction-related biomarkers may indicate, for example, poor outcomes for Covid-19 infected individuals due to a reactivation of the liver disease [1]. Furthermore, a reciprocal interrelation between liver diseases and infection by Covid-19 is proposed by several research groups as an severity level of both; hypoxia-, inflammation- and detoxification-related local and systemic impairments are involved in the pathogenesis [2–5].

Since 2009, a rapid rise in deaths due to liver diseases has been recorded in the USA especially amongst young people. Deaths due to cirrhosis increased by 65% and deaths due to liver cancer doubled in years 1999 to 2016 [6]. This trend carries a global character. For example, for South Korea, lung and liver cancers are predicted to be the most common malignancies which people will die from 2020 onwards [7].

Nowadays, liver disease accounts for approximately 2 million deaths per year worldwide: 1 million due to complications of cirrhosis and 1 million due to viral hepatitis and hepatocellular carcinoma [8]. The corresponding economic burden imposed on societies is enormous: Just non-alcoholic fatty liver disease alone affects roughly 100 million Americans and costs the United States healthcare system $32 billion annually [9].

Worldwide, more than half a million new cases of hepatocellular carcinoma (HCC) are diagnosed annually. Disease prognosis remains poor, with a 5-year survival rate across all disease stages estimated between 10% and 20%, and 3% for those diagnosed with distant disease [10]. The economic and healthcare burden of HCC is substantial. Patients need more effective therapeutic modalities prolonging survival and increasing the quality of life (QOL). Healthcare payers need to balance the cost-efficacy and QOL implication. To this end, additional research is needed to gain understanding of all aspects related to HCC management [10].

### Almost all solid tumours are capable to spread metastases to the liver

Besides primary liver malignancies, almost all solid tumours are capable to spread metastases to the liver, in particular gastrointestinal cancers, breast and genitourinary cancers, lung cancer, melanomas and sarcomas [11]. Therefore, the local liver-specific architecture as well as systemic effects such as ischemic-hypoxic niches and chronic inflammation characteristic even for young individuals with disturbed microcirculation (e.g. Flammer syndrome phenotype, amongst others) may play a crucial role in creating a “fertile” microenvironment which is highly supportive for metastatic spread to the liver [12, 13]. In turn, the properties of this microenvironment strongly depend on specific features of local and systemic molecular alterations which could be particularly opportune for primary and secondary tumour progression such as mitochondrial dysfunction, low energy supply, “Warburg” effect and chronic inflammation, amongst others [14].

As reported recently [15], systemic molecular set-up detected by the liquid biopsy approach might be highly indicative for individual outcomes under the palliative treatment of liver malignancies.

### Multiparametric set-up to model predictive and prognostic approach under the palliative treatment of liver malignancies

As detailed above, several million people currently suffer from primary and secondary liver malignancies—many of them undergo a palliative treatment due to the advanced stage of the disease. In order to improve individual outcomes as well as the cost-efficacy of the medical care, as precise as possible, stratification of patients is needed. Liquid biopsy is a promising approach to evaluate systemic effects and to provide comprehensive information for multiparametric analysis [16]. Furthermore, specific multi-omic patterns have been demonstrated to play a crucial role in cancer research and clinically relevant outcomes [17, 18]. For complex clinical situations, multiparametric disease modelling is essential to maximise the predictive power of diagnostic tools as demonstrated for multi-factorial diseases such as premenopausal breast cancer with high metastatic potential [19, 20].

### Working hypothesis

An evidence-based approach has been taken to target molecular pathways and subcellular components for modelling most optimal conditions with the longest survival rates for patients diagnosed with advanced liver malignancies who underwent palliative treatments. The following multitude diagnostic levels and pathways have been considered for the current study:

- Comprehensive patients’ data including the type of liver malignancy, treatment (SIRT versus TACE) and survival period of time after the treatment
- Systemic effects reflected in subcellular and molecular patterns of circulating leucocytes and blood serum
- Health status of leucocytes by “comet assay” imaging
- Expression patterns of SOD-2 and catalase—key-enzymes of the detoxification pathway
- Expression patterns of thioredoxin—the natural scavenger essential for any living organism
- Activities of metalloproteinases 2 and 9—key enzymes in tissue remodelling
- Expression patterns of calgranulin A (S100), GTPase Rho A and profilin 1—the tumour progression relevant proteins

To verify this hypothesis, *Unsupervised Machine Learning* approach has been applied.

## Materials and methods

### Recruitment of patients with primary hepatocellular carcinoma (HCC) and secondary hepatic metastases

This study was designed as a “pilot study” for the identification of a multi-level biomarker screening panel for patients with primary and metastatic liver malignancies who would be undergoing selective internal radiation therapy (SIRT) or transarterial chemoembolisation (TACE). Therefore, a wide range of malignancies of different types were incorporated in the study. The blood tests for the screening panels were performed prior to SIRT or TACE.

In total, 108 patients were considered for the study.

#### Inclusion criteria

- Primary hepatocellular carcinoma (38 patients)
- Metastases to the liver (70 patients)
- Treatment by SIRT (86 patients)
- Treatment by TACE (22 patients)

#### Exclusion criteria

- Pregnancy
- Acute infections (but not chronic hepatitis)
- Alcohol abuse
- Genetic disorders and disorders with premature ageing (Down Syndrome, Werner Syndrome, Alzheimer’s disease, others)

All the patients were informed about the purposes of the study and consequently have signed their “consent of the patient”. All investigations conformed to the principles outlined in the Declaration of Helsinki and were performed with permission by the responsible Ethics Committee of the Medical Faculty, Rheinische Friedrich-Wilhelms-Universität Bonn. Corresponding reference number is 283/10.

### Liquid biopsy: Blood samples collection, biobanking and biopreservation

Blood samples (20 ml) anti-coagulated with heparin were collected from the patients prior to any treatment application.

#### Biobanking

Both peripheral leukocytes and blood serum were separated and stored for all follow-up analyses.

Peripheral leukocytes were isolated using Ficoll-histopaque gradients (Histopaque 1077, Sigma, USA) as described elsewhere [21]. Briefly, blood samples were diluted with equal volumes of physiological buffer solution (PBS, Biochrom AG, Germany). Then, 2 ml of histopaque were placed into 10-ml sterile centrifuge tubes and 5 ml of diluted blood samples were carefully layered onto each histopaque gradient. Gradients were centrifuged at 475 g and 20 °C for 15 min. The leukocytes bands were removed from the interface between the plasma and histopaque layers of each tube and collected into one 50-ml tube. The total volume was brought to 50 ml with cold Dulbecco’s Modified Eagle Medium (DMEM, Gibco, USA). The cell suspension was washed three times with PBS and the total number of cells was determined.

Blood serum (500 μl) was separated by centrifugation from each blood samples not later than within 1 h after individual blood draw.

#### Biopreservation

Blood serum was frozen and stored at − 80 °C directly after each individual blood sample centrifugation. Separated peripheral leukocytes were finally re-suspended in PBS-DMSO solution, aliquoted into Eppendorf tubes and stored at − 80 °C until molecular profiling has been performed.

### Multi-omic analysis

#### Protein expression analysis by Western blotting

All analyses were performed two times for each sample utilising the standardised procedure described elsewhere [22]. Primary antibody incubation was performed at room temperature using a 1:200 dilution of the specific antibodies to

- Human calgranulin A, a goat polyclonal antibody (C-19) raised against a peptide mapping at the C-terminus of calgranulin A of human origin, *sc-8112*, Santa Cruz, USA
- Human catalase, a goat polyclonal antibody (S-20) raised against a peptide mapping an internal region of catalase of human origin, sc-34282, Santa Cruz, USA)
- Human profilin 1, a goat polyclonal antibody (C-15) raised against a peptide mapping at the C-terminus of profilin 1 of human origin, sc-30522, Santa Cruz, USA
- Human RhoA, a mouse monoclonal antibody (26C4) raised against an epitope corresponding to amino acids 120–150 of RhoA of human origin, sc-418, Santa Cruz, USA
- Human superoxide-dismutase (SOD-2), a goat polyclonal antibody (N-20) raised against a peptide mapping near the N-terminus of SOD-2 of human origin, *sc-18503*, Santa Cruz, USA
- Human thioredoxin (Trx), a mouse monoclonal antibody (D-4) specific for an epitope mapping between amino acids 1–34 at the N-terminus of Trx of human origin, sc-271281, Santa Cruz, USA.
- The house-keeping protein—human actin, a goat polyclonal IgG (I-19), epitope mapping at the C-terminus of actin of human origin, recommended for detection of a broad range of actin isoforms of human origin, *sc-1616*, Santa Cruz, USA.

The protein-specific signals were measured densitometrically using the Quantity One^®^ imaging system (Bio-Rad, USA).

#### Analysis of metalloproteinase activity by zymography

For determination of gelatinase activity of MMP-2 and MMP-9 in blood serum “Ready-Gelatin-Gels” (Bio-Rad, USA) were used according to the instructions of the manufacturer and as published earlier [22]. Two microliters from individual serum samples were electrophoresed under non-reducing conditions using Criterion™ Precast Gel System (Bio-Rad, USA). After electrophoresis, each gel was incubated at room temperature in 2% Triton X-100 for 2 × 30 min in order to remove the traces of sodium dodecyl sulphate, and then incubated overnight at 37 °C in buffer (150-mM NaCl, 50-mM Tris-HCl, pH 7.6, containing 5-mM CaCl_2_ and 0.02% NaN_3_). Afterwards a staining with 0.5% Coomassie blue G-250 (Sigma, USA) was performed for each gel. The proteolytic activity of each gelatinase (A and B) was identified as a clear band on a blue background according to the correspondent molecular weight of each gelatinase (A and B that corresponds to the metallproteinase-2 and -9, respectively). Gels were dried between cellophane sheets with a GelAir™ drying system (Bio-Rad, USA) and then scanned with a yellow filter using Adobe Photoshop (Adobe System, USA) in grey-scale mode. Densitometric analysis of zymographic lysis zones at 66 and 86 kDa for gelatinases A and B, respectively, was performed using Quantity One imaging system (Bio-Rad, USA).

#### Subcellular imaging: “comet assay” analysis of DNA fragmentation

In order to evaluate DNA quality (DNA damage)‚ the subcellular imaging by “comet assay” (Trevigen, Inc., Cat. No. 4250-050-K, USA) analysis has been used. ™The single cell gel electrophoresis assay is based upon the ability of DNA fragments to migrate out of the peripheral leukocytes in the electric field applied, whereas undamaged chromosomal DNA does not migrate into the agarose gel. DNA fragmentation assessment has been performed by evaluation of the DNA “comet” tail shape and specific migration patterns. Peripheral leukocytes have been immobilised in a bed of low melting point agarose, on a Trevigen CometSlide™. The alkaline electrophoresis is very sensitive and detects small amounts of damage. Therefore, after cell lysis, samples have been treated with alkali to denature the DNA and hydrolyse sites of damage. After electrophoretic separation, staining with a fluorescent DNA intercalating dye (SYBR® Green I) has been performed. The shape of individual “comets” has been visualised by epifluorescence microscopy. The evaluation system developed by the authors and published earlier [15] has been applied for the qualification and quantification of the DNA fragmentation/damage.

### Unsupervised machine learning (UML)

A Cox regression model was fitted independently for each biomarker while correcting for the confounders age, gender, therapy and primary tumour. An interaction effect of biomarker with therapy and primary tumour was included. A step-wise regression was used for model selection via the Akaike Information Criterion. The Cox proportional hazard assumption was tested in every case [23] and was never rejected. The entire analysis was carried out via R-package “survival”.

**Fig. 1:**
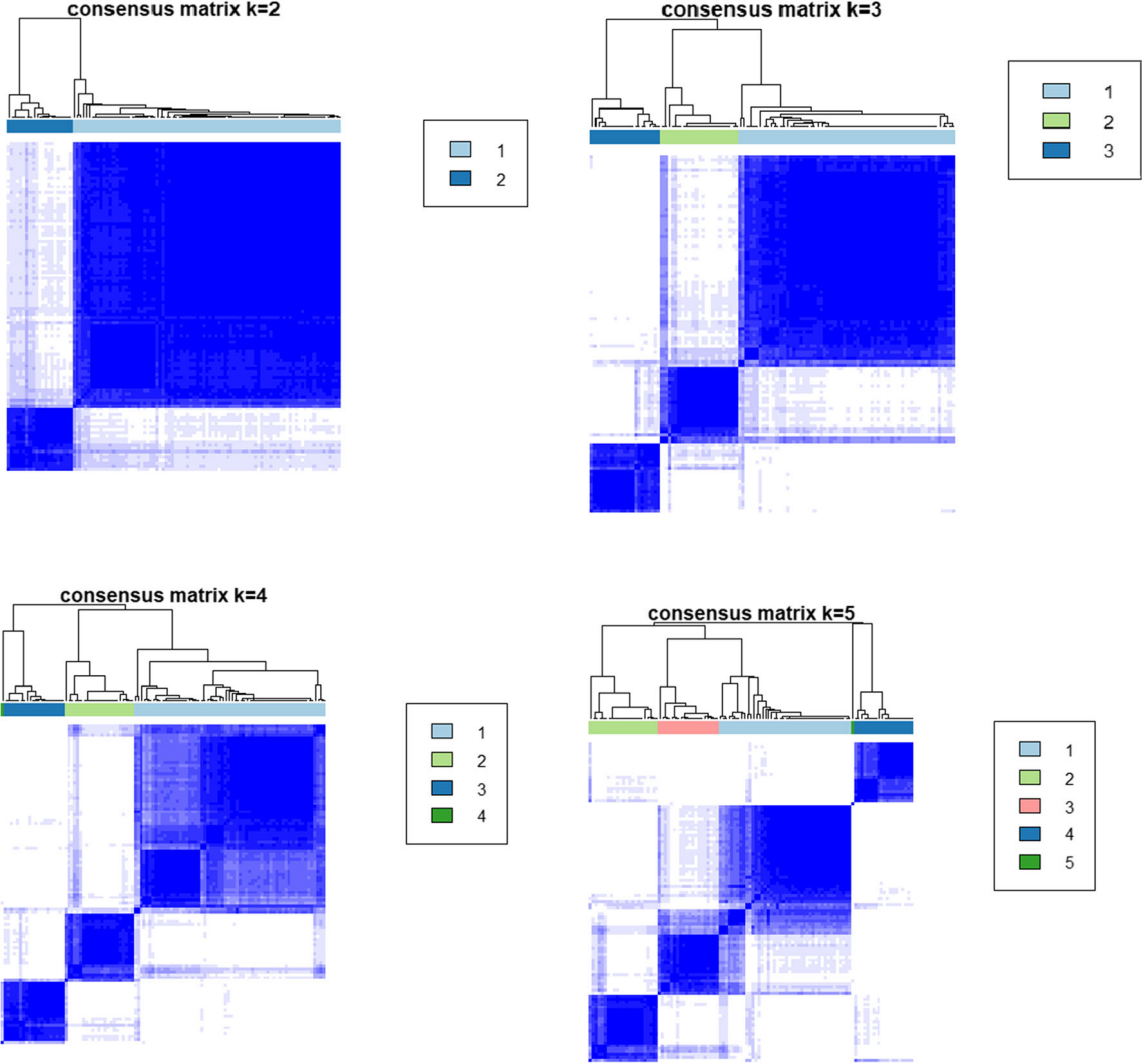
Heatmaps of consensus matrices for k = 2,3,4,5; rows and columns of consensus matrices correspond to individual patients involved into the study; consensus values range from 0 (white = not clustered together) to 1 (dark blue = always clustered together); hierarchical clustering of consensus matrices is depicted as dendrogram

In a second step, linear predictors of each Cox model were concatenated for every patient, resulting into an individual’s risk profile. Consensus hierarchical clustering (R-package “ConsensusClusterPlus”) was applied with 100 repeats for k = 2, 3, 4, 5 clusters (see Fig. 1). The optimal number of clusters was identified via the delta area under curve method [24]. The robustness of the clustering was checked via the consensus matrix.

The association of clusters with the confounders gender, therapy, disease and comorbidities was tested via a *χ*^2^-test. Differences of age across clusters were tested via an ANOVA F-test.

Pairwise differences of biomarkers between clusters were assessed via Wilcoxon’s rank test. *P* values were corrected for multiple testing via the Benjamini-Hochberg method [25].

## Results

We developed an unsupervised machine learning (UML) approach to robustly identify patient subgroups based on estimated survival curves for each individual patient and each individual potential biomarker. Since we investigated a panel of candidate biomarkers, each patient was effectively represented by a risk profile, and this risk profile was statistically corrected for confounding factors such as age, gender, therapy and primary tumour. In a second step, we then clustered patients based on their corrected risk profiles. This was done via consensus hierarchical clustering [24], which relies on repeatedly resampling and re-clustering patients to ensure a robust and stable grouping. Patient subgroups were subsequently once more investigated for confounders and for differences in each of the tested biomarkers.

**Fig. 2:**
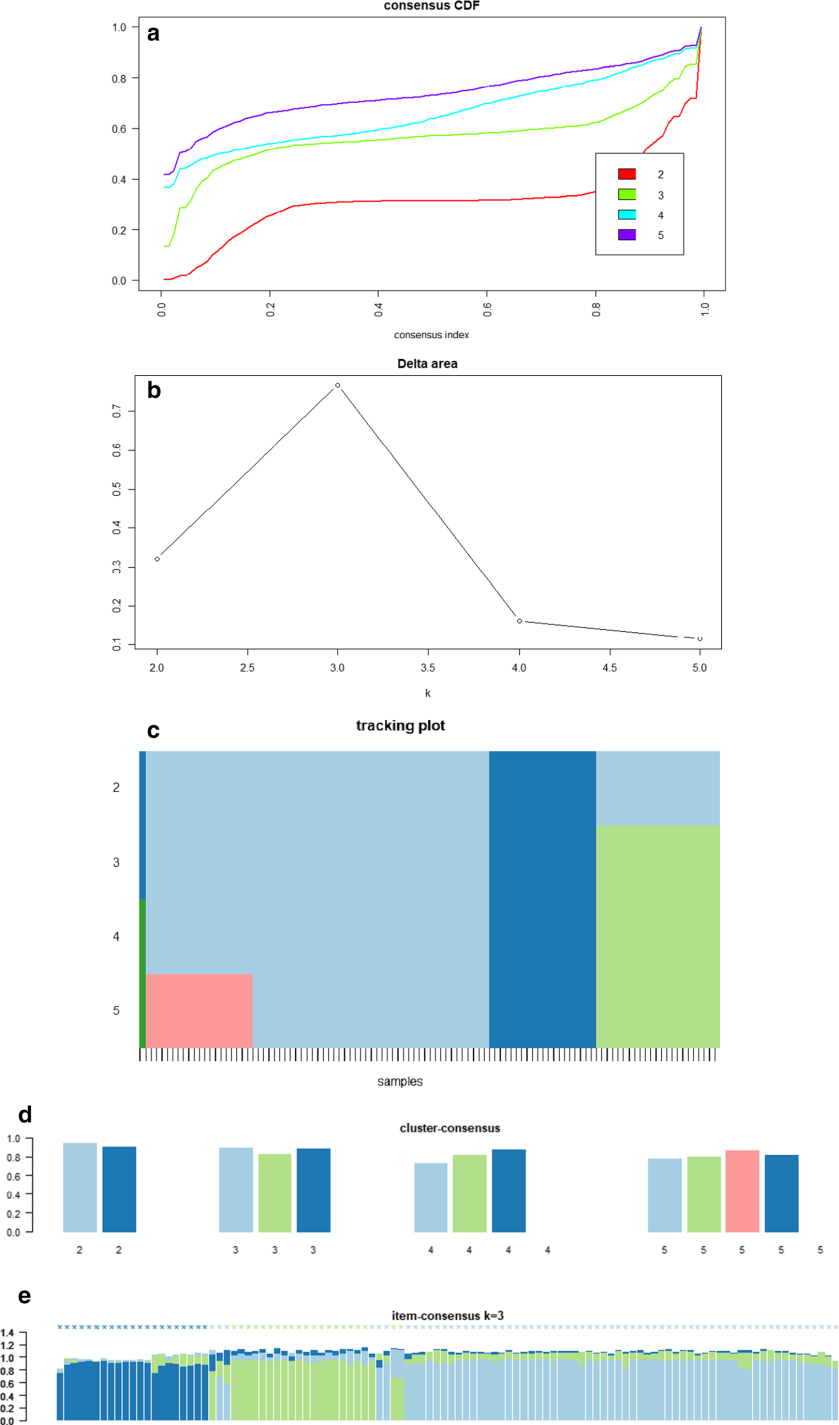
**a** Consensus Cumulative Distribution Function (CDF) of entries in the consensus matrix; the ***y***-axis is the cumulative distribution function, whereas the ***x***-axis is the consensus value in the consensus matrix. **b** Delta area plot highlighting the change of the area under the CDF curve; the strongest increase in this area can be observed for k = 3 clusters. **c** Tracking plot showing the cluster assignment of patients indicated as columns for different choice of number of clusters k (as rows); colours indicate clusters; hatch marks below the plot indicate patients; thereby, patients frequently changing colours within a column are indicative for unstable cluster membership; herewith, no unstable membership can be recognised in the presented plot. **d** Cluster consensus plot showing the mean consensus value of patients assigned to a defined cluster; colours are in agreement to the tracking plot; high values indicate high stability of a given cluster; in contrast, low values indicate instability of a given cluster: For k = 3, all clusters demonstrate high stability. **e** Item-consensus plot showing the mean consensus of all patients within k = 3 clusters; consensus values are indicated by the heights of bar; they correspond to the fraction of times that a dedicated patient shown on the ***x***-axis was assigned to a cluster indicated by its colour; the colouring scheme is in agreement to the previous figures; asterisks on the top of each bar indicate the consensus cluster for each patient; in summary, this plot enables to recognise whether a patient is a “pure” member of a cluster or whether it shares a high consensus to multiple other clusters (indicated by multiple coloured bars of equal sizes); it can be observed, therefore, that most of patients are “pure” members of one of the 3 clusters

**Fig. 3:**
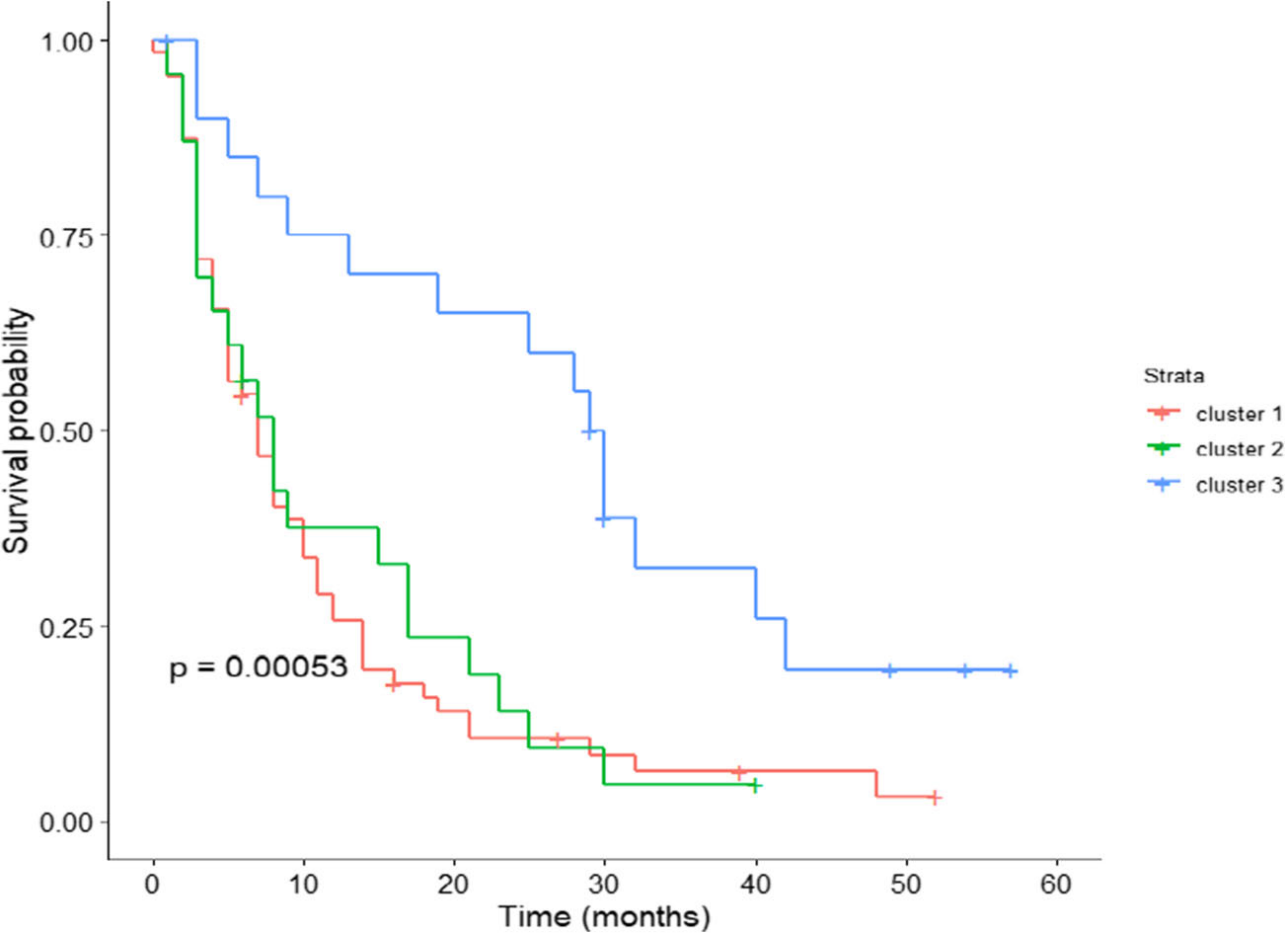
Kaplan-Meier curves (overall survival) of patients stratified into 3 clusters by consensus clustering; small vertical tick-marks indicate right censored survival times of individual patients; log-rank test was used to estimate the *P* value

**Fig. 4:**
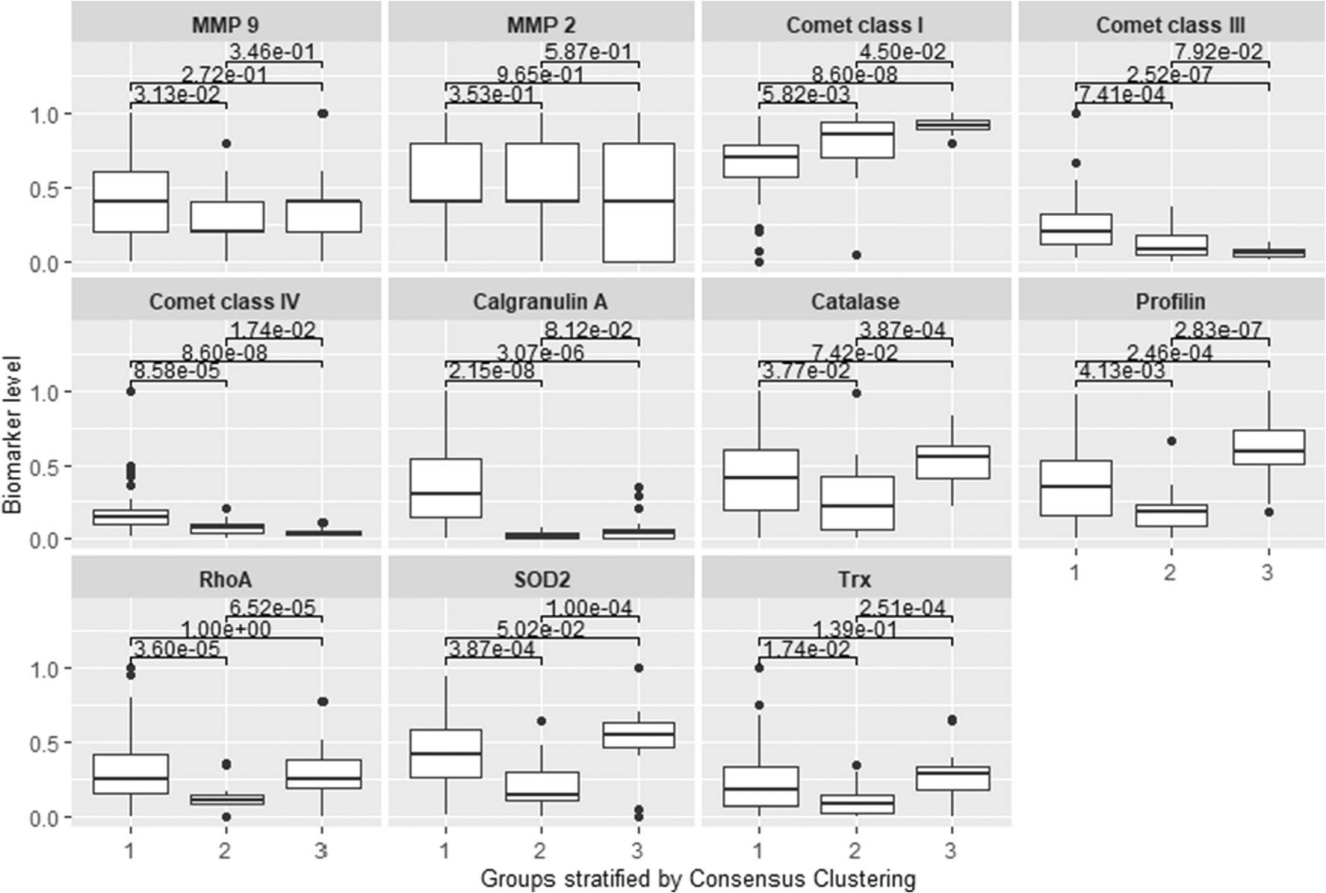
Biomarker patterns recorded for stratified patient groups by the consensus clustering method (see Fig. 3 for corresponding survival rates); all biomarker values were normalised between 0 and 1; pairwise differences of biomarkers between clusters were assessed via Wilcoxon’s rank test with correction for multiple testing via the Benjamini-Hochberg method; patterns demonstrating high values of “comet assay” class I and low values of “comet assay” classes III and IV and calgranulin expression correspond with best survival rates

The consensus clustering method involves subsampling from a subgroup of patients and determines clustering of specified number of clusters (k) (Fig. 1). Consensus hierarchical clustering of biomarker-derived risk profiles resulted into 3 stable patient subgroups (Figs. 1, 2, and 3). There were no significant differences in age, gender, therapy, diagnosis or comorbidities across clusters. Survival times across clusters differed significantly (log-rank test, Fig. 3). Furthermore, several of the biomarkers demonstrated highly significant pairwise differences between clusters after correction for multiple testing (Fig. 4), namely, “comet assay” classes I, III and IV; calgranulin A; SOD2; and profilin. Hence, these markers can be viewed as promising candidates for predicting differences in the prognostic outcome of patients. As next steps we thus recommend a further retrospective validation in another study and finally a prospective clinical trial to confirm the prognostic value of our candidate biomarker signature. Provided that such a validation is positive, the development of a diagnostic test for clinical routine should be considered. This diagnostic test would have to be approved by regulatory agencies, such as the EMA in Europe and the FDA in the USA. In that context, one has to judge the costs of the test in comparison with the actual added value for the patient.

### Data interpretation, conclusions and expert recommendations in the context of 3P medicine

#### The principle conclusion

In our model, the multiparametric profiles demonstrate a highly significant (*P* = 3e-04) difference between the gradients characteristic for worst (Kaplan-Meier curve 1, Fig. 3) and intermediate (Kaplan-Meier curve 2, Fig. 3) versus the best (Kaplan-Meier curve 3, Fig. 3) survivals within 5 years of observations after corresponding treatments. Thereby, a particularly strong difference between the survival profiles was demonstrated for the first 30 months after treatments (Fig. 3). Therefore, the principle conclusion made was that parameters chosen for the analysis are highly relevant for predicting survivals under the study conditions.

#### Critical evaluation of contributing parameters in the model

From altogether 11 molecular and subcellular parameters used for the study, six parameters demonstrated significant differences in corresponding values, when the worst and best survival curves are compared (Fig. 4), namely:

- “Comet assay” patterns of class I, III and IV
- Expression patterns of Calgranulin A (S100)
- Expression patterns of SOD-2
- Expression patterns of profilin

Corresponding functions of the above-listed pathways relevant for the tumour progression have been described elsewhere. Therefore, a detailed discussion on the matter is turned down with the reference to previous publications [15].

Considering worst, intermediate and best survival curves (1, 2 and 3, respectively, in Fig. 3) with regard to corresponding patterns of parameters measured (Fig. 4), the most convincing images are presented by “comet assay” patterns of class I, III and IV and expression patterns of S100. All four images in Fig. 4 demonstrate clear gradients of corresponding parameter values for the worst, intermediate and best survivals in the model. With other words, these four parameters can be applied as are for the clinical validation of the predictive diagnostic approach.

More complex is the situation when the remaining five parameters (of altogether 11 parameters, see Fig. 4), are considered. In this context, we would like to refer to our previously published article evaluating GTPase Rho A and MMP-9 as potential biomarkers, individually and in set, in breast cancer prediction. This study has clearly demonstrated that only when both biomarkers were applied in set, a detailed patient stratification could be achieved [22, 26].

#### Study limitations and a strong potential for the model’s improvement

**Fig. 5:**
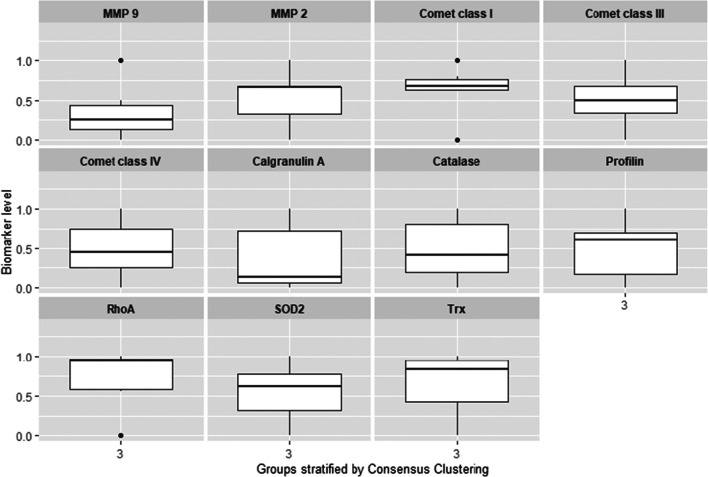
Biomarker patterns recorded for the stratified group of six patients belonging to the cluster 3 (altogether 22 patients) demonstrating the best survival rates (see Fig. 3); these six patients were diagnosed with HCC and underwent TACE; although biomarker patterns follow general trends demonstrated in Fig. 4, the group-specific difference is evident, demonstrating lower median value for MMP-9 activities and higher median values for “comet assay” classes III and IV, S100, RhoA and thioredoxin compared with these of non-stratified 108 patients involved in the study

Certainly, the main limitation of the study is a small number of patients with highly heterogeneous individual patient profiles such as original malignancy diagnosed, gender and the therapeutic approach (SIRT versus TACE). Figure 5 illustrates this deficit on the example of six patients who were diagnosed with HCC and underwent TACE being included into the group of best survivors presented as cluster 3 in Fig. 3. Altogether, these are 22 patients in cluster 3 diagnosed either with HCC (9 patients who underwent either SIRT or TACE) or colorectal cancer (9 patients who underwent SIRT) and breast cancer (4 patients who underwent SIRT). For six patients originally diagnosed with HCC and underwent TACE, it can be easily recognised that, although their biomarker patterns follow general trends demonstrated for cluster 3 in Fig. 4, there is a group-specific difference such as

- Lower median value for MMP-9 activities
- Higher median values for “comet assay” classes III and IV, S100, RhoA and thioredoxin

Consequently, more detailed patient stratification is need in bigger patient groups including age, gender, detailed description of collateral pathologies and treatments applied. Further, clustering of biomarker sets is essential as stated above on the example of GTPase Rho A and MMP-9 [22]. It is obvious that synergic and compensatory effects may play a crucial role in the overall balance of systemic effects and individual molecular pathways such as scavenger activities (high level of thioredoxin expression), detoxification capacity (SOD-2 and Catalase interplay), immune system capacity (intact leucocytes reflected in class I of the “comet assay” patterns) and modulation of metastatic potential, amongst others [12, 15, 20, 27].

### What is this model good for? Expert recommendations in the context of 3P medicine

#### Immune system

Patterns of circulating leucocytes are the most relevant biomarkers in our model. Obviously survival rates are directly dependent on the DNA quality demonstrated by “comet assays” in leucocytes: more healthy cells—higher survival chances in all patient subgroups involved. This result is highly relevant for disease monitoring, disease prognosis and potential therapy modalities focused on the immune system function.

#### Metastatic potential

Another highly relevant biomarker in our model is S100: Low expression levels of S100 correlated with the best survival rates. Noteworthy, in our model, S100 was measured in circulating leucocytes that emphasises the systemic effects by S100. S100 is known to be instrumental for cancer development and progression. Contextually, it is highly recommended to involve this biomarker into routine cancer-related predictive diagnostics and disease monitoring.

#### Detoxification and scavenging activity

SOD-2 expression in circulating leucocytes was significantly increased in patients with the best survival rates. SOD-2 function is fundamental for detoxification pathways and scavenging activities in the human body. Therefore, it is highly recommended to involve this biomarker into routine cancer-related predictive diagnostics and disease monitoring. Complementary information can be received by measurements of thioredoxin levels (see Fig. 5) known as a highly potent scavenger and redox-control-based regulator of central biological processes such as immune system functioning, stress response, DNA synthesis de novo, regulation of transcriptome, and restoration of protein redox status with corresponding activities, amongst others. Overall scavenging activities in cancer management is an attractive topic to be consulted with dietologists based on individualised patient profiling to recommend appropriate nutrients and dietary supplements. In particular, patient-adapted levels of scavenger activities should be taken into consideration due to their genoprotective effects. However, particularly in palliative care, the quantity and quality of the genoprotective and scavenging dietary supplement should be carefully considered distinguishing between the needs of functional tissue such as immune system on one hand and biological process specific for progressing metastatic disease on the other hand [14, 28]. To this end, cytotoxic effects suppressing specifically circulating tumour cells and metastatic disease have been demonstrated for some herbs and their constituent phytochemicals such as Apigenin and natural polyphenol Calebin A which per evidence suppress proliferation, invasion and metastatic spread [29, 30]. Corresponding mechanisms involve NF-κB-related signalling pathways, SOD-2 and thioredoxin system activities, and ROS-inhibition with redox-based therapeutic effects [29, 31].

In conclusion, the paradigm of the palliative care in the liver cancer management is evolving from “just end of the life” care to careful evaluation of all aspects relevant for the survivorship [28]. In light of the above, a multi-faceted cancer control within palliative care is crucial for improved disease outcomes including individualised patient profiling, predictive modelling and corresponding cost-effective risks mitigating measures. For this purpose, a multi-omic approach is crucial for reliable prediction [17].

Our multiparametric approach for a detailed patient stratification in palliative treatment of liver malignancies follows concepts of cost-effective and patient-centred care [32]. Conclusions presented in this article conform with 3 PM principles constituted by the European Association for Predictive, Preventive and Personalised Medicine which provides a clear benefit to the patient and healthcare as a whole [33]. At this point, we would like to mention again that before implementation into clinical routine a prospective study for validation of the stratification presented in this article would be required. Provided that such a validation is positive, the development of a diagnostic test for clinical routine should be considered. This diagnostic test would have to be approved by regulatory agencies, such as the EMA in Europe and the FDA in the USA.
